# Supplementary material for: The effect of military training on the sense of agency and outcome processing
Source: Nat Commun. 2020 Aug 31;11:4366. doi: 10.1038/s41467-020-18152-x (PMC7459288; doi:10.1038/s41467-020-18152-x)
Supplement: Supplementary file 1 — Supplementary Information [file 41467_2020_18152_MOESM1_ESM.pdf]

Supplementary Information for

The effect of military training on the sense of agency and outcome processing

Caspar, et al.

Correspondence to: ecaspar@ulb.ac.be

SUPPLEMENTARY NOTES 1

Number of shocks (Study 1). On average, participants administered 31.26/60 (SD=13.58, min: 0 – max: 60) shocks to the 'victim' in the free-choice condition. We conducted a factorial ANOVA with the number of shocks that agents freely delivered as the dependent variable and Group (junior cadets vs. civilian students) and Experimenter (ranked experimenter vs. civilian experimenter) as fixed factors. Neither the main effect of Group (p>.9), nor the main effect of Experimenter (p>.6), nor their interaction (p>.8) influenced the number of shocks freely administered by the agents.

Vindictive behavior (Study 1). Because of role reversal in the present experiment, we could investigate whether experiencing pain first as a 'victim' guided subsequent choices regarding inflicting shocks on one's co-participant. We conducted a linear regression with the number of shocks received when 'victim' as the independent variable and the number of shocks freely delivered when 'agent' as the dependent variable. This analysis was conducted on those participants who were 'victims' first. We found evidence for a positive relation, indicating vindictive behavior, in the civilian students group with both the ranked experimenter (t(9)=3.765, p=.006, Beta=.800) and the civilian experimenter (t(9)=2.852, p=.021, Beta = .710). Specifically, the more shocks participants received when they were 'victims', the more shocks they administered when they were agents. For the junior cadets group, we found no evidences for such vindictive behaviors, neither with the ranked experimenter (p>.1), nor with the civilian experimenter (p>.6).

SUPPLEMENTARY NOTES 2

Responsibility ratings (Study 1). We conducted a repeated-measures ANOVA on agents' explicit ratings of their feeling of responsibility, with Condition (free-choice vs. coercion) as within-subjects factor and Group (junior cadets vs. civilian students), Experimenter (ranked experimenter vs. civilian experimenter) and Role Order (agent first vs 'victim' first) as between-subject factors. As expected, we observed a main effect of Condition (F(1,68)=167.1, p<.001, $\eta^2_{partial}$ =.711), with higher responsibility ratings in the free-choice condition (89%, CI_{95}=86-92) when compared to the coercion condition (41.5%, CI_{95}=34-49). This effect was influenced neither by the Group (p>.1), nor by the Experimenter (p>.8), nor by the Order of the role (p>.6). Other main effects and interactions were not significant (all ps>.3).

SUPPLEMENTARY NOTES 3

Predisposition trait to join military organizations. First, we compared scores on different personality questionnaires between the group of junior cadets and our control group of civilians to explore whether any trait differences could account for the results observed. We observed that junior cadets scored higher on the SDO scale (mean score 53, SD=12.56) than civilian students (mean score: 42, SD=17.22, t(71)=-3.158, p = .002). Junior cadets also scored lower on the Levenson impulsivity subscale (mean score: 19, SD=4.34) than civilian students (mean score: 17, SD=3.17, t(71)=2.008, p = .048). To assess whether these trait differences might contribute to our main result, we predicted the coercion effect (defined as the difference in interval estimates between free choice and coercion conditions) against the SDO and Levenson scores in two separate linear regressions, using the full sample of participants from both groups. Neither personality measure predicted the coercion effect (ps

> .08). We performed similar regressions for the coercion condition alone and for the free-choice condition alone, and again failed to find significant relation (*p*s > .1).
We additionally performed Pearson correlations between the z scores in both the free-choice and the coercion conditions for the civilians and the 3 additional questions that we asked after the experimental session. Those questions were designed to identify counterfactual predispositions to join military organizations within the non-military control group. Of note, since it was a post-session questionnaire, only 32/40 participants responded. Within the control group, there was no evidence for a relation between the coercion effect and the response to any of these questions (all *p*s > .1). Similar analyses of the free-choice or the coercion condition also failed to show significant effects (all *p*s>.3).

SUPPLEMENTARY NOTES 4

Number of shocks (Study 2). On average, participants administered 33.38/60 (SD=15.65, min: 0 – max: 60) shocks to the 'victim' in the free-choice condition. A factorial ANOVA with the number of shocks that agents freely delivered as the dependent variable and Group (junior cadets vs. seniors vs. privates) and Order of the Role (agent first vs. 'victim' first) as fixed factors. Neither the main effect of Group ($p > .1$), nor the main effect of Order of the Role ($p > .7$), nor their interaction ($p > .6$) influenced the number of shocks freely administered by the agents.
Vindictive behaviors (Study 2). We conducted a linear regression with the number of shocks received when 'victim' as the independent variable and the number of shocks freely delivered when 'agent' as the dependent variable. Such as in Study 1, this analysis was conducted on those participants who were 'victims' first. We found evidence for a positive relation, indicating vindictive behavior, in the three groups (junior cadets: t(14)=2.455, $p = .029$, Beta=.563 – seniors: t(14)=6.925, $p < .001$, Beta=.887 – privates: t(14)=6.008, $p < .001$, Beta=.857).

SUPPLEMENTARY NOTES 5

Responsibility ratings (Study 2). We conducted a repeated-measures ANOVA on agents' explicit rating on their feeling of responsibility, with Condition (free-choice vs. coercion) as within-subject factor and Group (junior cadets vs. seniors vs. privates), and Order of the Role (agent first vs. 'victim' first) as between-subject factors. We observed a significant effect of Condition (F(1,78)=20.509, $p < .001$, $\eta^2_{partial} = .208$), with higher responsibility ratings in the free-choice condition (87%, CI_{95}=83-90) than in the coercion condition (49%, CI_{95}=32-66). None of the other main effects or interactions were significant (all *p*s > .1).

SUPPLEMENTARY NOTES 6

Supplementary analysis to investigate a potential effect of selection process. On the 66 junior cadets tested in both Study 1 and Study 2, 19 did not persevere in the military system after their first year (i.e. group OUT). Their mean age was 19.47 (SD=3.27) and did not significantly differ from the mean age of the cadets who belong to the group IN (18.84, SD=1.106, $p > .4$). We conducted two repeated-measures ANOVA with Condition (Free-choice, Coercion) as within-subject factor and Group (IN, OUT) as between-subject factor on interval estimates and on the amplitude of the auditory N1.

Interval estimates. We observed that neither the main effect of Condition ($p > .1$), nor the main effect of Group ($p > .8$), nor their interaction ($p > .6$) influenced interval estimates. Since we previously observed that sense of agency differed between groups in the free-choice condition only, supplementary independent sample t-tests conducted on the free-choice condition indicated that z scores were higher for the group of cadets who persevered in the military system (z scores: -.031, CI_{95}= -.081 – .018) than for the group of senior cadets (t(77)=2.157, p = .034, Cohen's d = .489), indicating a lower sense of agency in cadets who persevered in the military system (see **Supplementary Figure 2**). Results did not differ in the free-choice condition between senior cadets and cadets who left the military system.

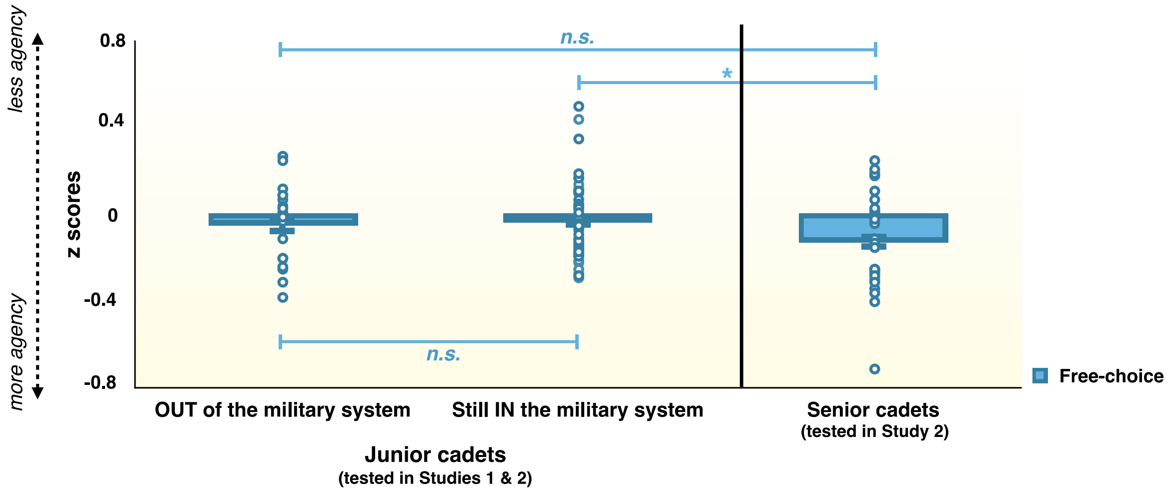

Supplementary Figure 1. z scores in the free-choice condition, respective to the Group (junior cadets OUT – n=19 vs junior cadets IN – n=47 vs senior cadets – n=30). Data are presented as mean values +/- SEM. Errors bars represent standard errors. Graphical display showing independent sample t-tests (blue significance lines) between the three groups. Errors bars represent standard errors. All tests were two-tailed. * represents a p value between .01 and .05. *n.s.* indicates a non-significant result. Source data are provided as a Source Data file.

EEG recordings. We observed a significant main effect of Condition (F(1,63)=18.674, p < .001, $\eta^2_{partial}$ = .229), with as expected a higher amplitude of the auditory N1 in the free-choice condition (-10.3 µv, CI_{95}=-11.35 – -9.26) than in the coercion condition (-8.14 µv, CI_{95}=-9.3 – -6.9). The main effect of Group was also significant (F(1,63)=7.794, p = .007, $\eta^2_{partial}$ = .110). The amplitude of the auditory was higher for the group OUT (-10.6 µv, CI_{95}=-12.2 – -8.9) than for the group IN (-7.85 µv, CI_{95}=-8.9 – -6.8), see **Supplementary Figure 2**. The interaction Condition x Group was not significant ($p > .5$). Independent samples t-tests confirmed that the OUT group had a higher amplitude of the auditory N1 in both the free-choice (t(63)=-2.943, p = .005, Cohen's d = .776) and the coercion condition (t(63)=-2.086, p = .041, Cohen's d = .534). It thus indicates that the amplitude of the auditory N1 was higher in both the free-choice and the coercion condition for the group of those who left the military system in comparison with those who persevere. Supplementary independent sample t-tests revealed that the pattern of results obtained for those who left the military system was similar to the one obtained by senior cadets ($p > .4$ in the free-choice condition and $p > .3$ in the coercion condition) and that those who stayed in the military system had a lower amplitude of the auditory N1 than senior cadets in both conditions (Free-choice: t(74)=2.321, p = .023, Cohen's d= .537 – Coercion : t(74)=3.728, p < .001, Cohen's d=.858). It thus confirmed that

the upregulation of outcome processing observed for senior cadets in Study 2 is not the effect of selection but rather an effect of training.

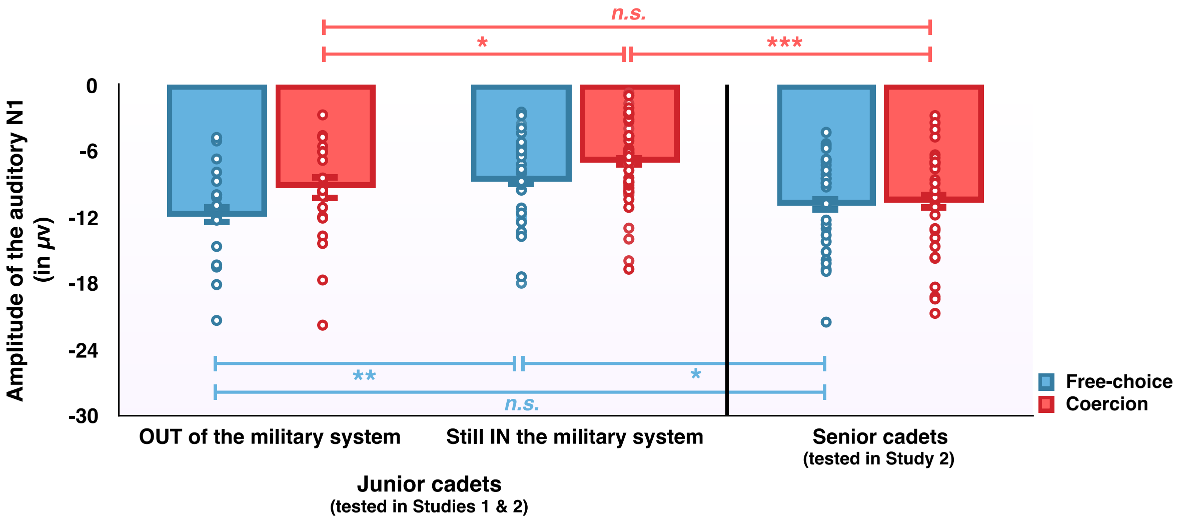

Supplementary Figure 2. Mean amplitude of the auditory N1 in both the free-choice (blue) and the coercion (red) conditions, respective to the Group (junior cadets OUT – n=19 vs junior cadets IN – n=47 vs senior cadets – n=30). Data are presented as mean values +/- SEM. Errors bars represent standard errors. Graphical display showing independent (blue, red significance lines) sample t-tests between the groups across experimental conditions. All tests were two-tailed. *** represents a p value < .001. ** represents a p value between .001 and .01. * represents a p value between .01 and .05. *n.s.* indicates a non-significant result. The group of junior cadets IN had a lower amplitude of the auditory N1 in the free-choice condition than the group of junior cadets OUT (p=.005) and, than the group of senior cadets (p=.023). The group of junior cadets IN also displayed a lower amplitude of the auditory N1 in the coercion condition than the group of junior cadets OUT (p=.041) and, than the group of senior cadets (p<.001). Source data are provided as a Source Data file

SUPPLEMENTARY NOTES 7

Withdrawal from the experiment. Two participants withdrew their participation in Study 1 and one participant did not complete the coercion condition as agent in Study 2. Their data were not taken into account in the statistical analysis. The data of their co-participant was included in the final sample. The first participant who withdrew his participation was in the civilian group tested by the civilian experimenter (1), the second one was in the junior cadet group tested by the ranked experimenter (2), and the third one in the group of privates.

(1) The first participant started as 'victim'. When he switched to the role of the agent, he started with the free-choice condition and decided not to administer shocks to the 'victim'. In the coercion condition, the first time that the experimenter told him to deliver a shock, he pressed the other key. The experimenter thus told him to follow the instruction that was given. On the second time that the experimenter ordered to give a shock to the victim, the participant reported feeling uncomfortable, but stated that he did not want to stop the experiment. However, we (the experimenter and the main investigator who were both present in the room) decided to stop the experiment. During the debriefing, he reported that he could not do to others something that he did not want to do to himself.

(2) The second participant was randomly assigned to start as the agent. After reading the

information sheet and listened to the instructions about the experiment, he said that he did not want to be the agent first but that the role of the 'victim' was suitable for him. The other participant agreed to start as the agent. When arrived the time of role reversal, he confirmed that he did not want to be in the role of the agent. During the debriefing, he reported that even if the shocks were not very painful, he felt uncomfortable with the idea to deliver a shock to the co-participant without any other reasons than the monetary reward.

(3) The third participant started as agent. He started with the free-choice condition and did not administer shocks to the 'victim'. In the coercion condition, the first time that the experimenter told him to deliver a shock, he pressed the other key. The experimenter thus told him to follow the instruction that was given. The next time the experimenter told him to deliver a shock, he again pressed the other button. We (the experimenter and the main investigator who were both present in the room) asked him if he wanted to continue this experimental condition and he told us not because he did not want to deliver a shock. He added that he did not want to stop the entire experiment and asked to continue as 'victim'. The other participant thus turned agent and followed instructions in the coercion condition. During the debriefing, he reported that he felt uncomfortable with the idea to deliver a shock to the co-participant in exchange for money.
