# Supplementary material for: The effect of military training on the sense of agency and outcome processing
Source: Nat Commun. 2020 Aug 31;11:4366. doi: 10.1038/s41467-020-18152-x (PMC7459288; doi:10.1038/s41467-020-18152-x)
Supplement: Supplementary file 3 — Reporting Summary [file 41467_2020_18152_MOESM3_ESM.pdf]

nature research

Corresponding author(s): Emilie A. Caspar

Last updated by author(s): Jun 10, 2020

Reporting Summary

Nature Research wishes to improve the reproducibility of the work that we publish. This form provides structure for consistency and transparency in reporting. For further information on Nature Research policies, see Authors & Referees and the Editorial Policy Checklist.

Statistics

For all statistical analyses, confirm that the following items are present in the figure legend, table legend, main text, or Methods section.

n/a	Confirmed	
☐	☒	The exact sample size (*n*) for each experimental group/condition, given as a discrete number and unit of measurement
☐	☒	A statement on whether measurements were taken from distinct samples or whether the same sample was measured repeatedly
☐	☒	The statistical test(s) used AND whether they are one- or two-sided<br>*Only common tests should be described solely by name; describe more complex techniques in the Methods section.*
☐	☒	A description of all covariates tested
☐	☒	A description of any assumptions or corrections, such as tests of normality and adjustment for multiple comparisons
☐	☒	A full description of the statistical parameters including central tendency (e.g. means) or other basic estimates (e.g. regression coefficient) AND variation (e.g. standard deviation) or associated estimates of uncertainty (e.g. confidence intervals)
☐	☒	For null hypothesis testing, the test statistic (e.g. *F*, *t*, *r*) with confidence intervals, effect sizes, degrees of freedom and *P* value noted<br>*Give P values as exact values whenever suitable.*
☐	☒	For Bayesian analysis, information on the choice of priors and Markov chain Monte Carlo settings
☒	☐	For hierarchical and complex designs, identification of the appropriate level for tests and full reporting of outcomes
☐	☒	Estimates of effect sizes (e.g. Cohen's *d*, Pearson's *r*), indicating how they were calculated

Our web collection on statistics for biologists contains articles on many of the points above.

Software and code

Policy information about availability of computer code

Data collection	MATLAB - Psychtoolbox for behavioral data & Biosemi - Actiview software for EEG data
Data analysis	MATLAB (version 2013A) and SPSS (version 22) for behavioural data & MATLAB Fieldtrip toolbox for EEG data

For manuscripts utilizing custom algorithms or software that are central to the research but not yet described in published literature, software must be made available to editors/reviewers. We strongly encourage code deposition in a community repository (e.g. GitHub). See the Nature Research guidelines for submitting code & software for further information.

Data

Policy information about availability of data

All manuscripts must include a data availability statement. This statement should provide the following information, where applicable:

- Accession codes, unique identifiers, or web links for publicly available datasets
- A list of figures that have associated raw data
- A description of any restrictions on data availability

Data are available on Open Science Framework (DOI 10.17605/OSF.IO/8U6HP) - direct link: https://osf.io/8u6hp/

Field-specific reporting

Please select the one below that is the best fit for your research. If you are not sure, read the appropriate sections before making your selection.

☐ Life sciences ☒ Behavioural & social sciences ☐ Ecological, evolutionary & environmental sciences

For a reference copy of the document with all sections, see nature.com/documents/nr-reporting-summary-flat.pdf

Behavioural & social sciences study design

All studies must disclose on these points even when the disclosure is negative.

Study description	We compared in Study 1 a group of civilian students to a group of junior cadets in order to investigate the effect of working in a military system on different quantitative data across two experimental conditions (i;e. free-choice and coercion). Quantitative data are behavioral (e.g. number of shocks delivered to the other participant, explicit responsibility ratings and time perception) and electrophysiological (ampitude of the auditory N1). In Study 2, we replicated the same experimental design than Study 1 but with three different groups of military staff: junior cadets, senior cadets and privates in order to evaluate how the military ranks influence the same quantitative data as in Study 1.
Research sample	In study 1, eighty naïve male participants were recruited in dyads. Forty of these participants were undergoing officer training at the Royal Military Academy of Belgium (RMA), while forty civilian participants were following standard university education, mainly at the Université libre de Bruxelles.<br>For Study 2, ninety new naïve male participants were recruited in dyads. Thirty were junior cadets (from RMA), thirty were seniors (from RMA) and thirty were privates (recruited on different military bases in Belgium).
Sampling strategy	Participants were selected based on voluntary participation within each group (civilians, junior cadets, senior cadets, privates).<br>To estimate the sample size, we used the effect size of Experiment 2 in [19]. In that study the effect size (dz) was 0.630 (based on the means and SDs of the within-subjects free-choice (mean: 367, SD: 119) and the coercive (mean: 426, SD: 131) conditions). To achieve a power of .80 for this effect size, the estimated sample size was 22 [15]. No previous studies tested the interaction of interest of the present study. We thus set the total sample size to 80 based on the fact that we had two main between-subject factors with two levels each and that junior cadets are limited in number. For Study 2, based on a similar method we increased the power up to .09 with N=30 per group (since for study 2 we did not include the betwen-subject factor 'type of experimenter'). The recruitement of participants in study 2 took 2 years because senior cadets are even more limited in number than junior cadets.
Data collection	Data collection was based on different methods.<br>For interval estimates, participants were given a pen and a paper sheet and they had to provide on each trial, a number between 1 and 1,000. These data were then associated with the output file from MATLAB in order to match them with the different independant variables. EEG data were recorded with a 32-channels Biosemi system in Study 2 and a 64-channels Biosemi system in Study 1. The main researcher who analysed the data was present in the room during each experimental session in order to manage the EEG recordings, but did not interact further with participants. Two other experimenters (i.e. the ranked officer or the civilian experimenter) run the experiments (description of the study, orders given in the coercion condition, etc.). It was systematically clear which condition (free-choice, coercion) was running for both the experimenter and the participants since verbal orders were given in the coercion condition and not in the free-choice condition.<br>The experimenter giving orders was systematically present in the room, as well as another experimenter who checked the quality of the EEG recordings.
Timing	For study 1, data collection for junior cadets and civilians were collected between October 2016 and February 2017. For Study 2, data collection took place between November 2017 and February 2019.
Data exclusions	The following exclusion criteria were determined prior to further analysis: failure to produce temporal intervals co-varying monotonically with actual action-tone interval, or failure to follow instructions. To identify participants for whom the action-tone intervals did not gradually increase with action-tone intervals, we performed a linear trend analysis with contrast coefficients -1, 0, 1 for the three delays we used. For Study 1, one participant was excluded due to a non-significant linear trend analysis. Two participants withdrew their participation during the experiment. For Study 2, based on the similar exclusion criteria than those we used in Study 1, three participants were excluded: Two participants were excluded due to a non-significant linear trend analysis. One participant withdrew his participation when he started the coercion condition as agent
Non-participation	Two participants withdrew their participation in Study 1 and one participant did not complete the coercion condition as agent in Study 2. Their data were not taken into account in the statistical analysis. The data of their co-participant was included in the final sample. The first participant who withdrew his participation was in the civilian group tested by the civilian experimenter (1), the second one was in the junior cadet group tested by the ranked experimenter (2), and the third one in the group of privates.<br><br>(1) The first participant started as 'victim'. When he switched to the role of the agent, he started with the free-choice condition and decided not to administer shocks to the 'victim'. In the coercion condition, the first time that the experimenter told him to deliver a shock, he pressed the other key. The experimenter thus told him to follow the instruction that was given. On the second time that the experimenter ordered to give a shock to the victim, the participant reported feeling uncomfortable, but stated that he did not want to stop the experiment. However, we (the experimenter and the main investigator who were both present in the room) decided to stop the experiment. During the debriefing, he reported that he could not do to others something that he did not want to do to himself.<br><br>(2) The second participant was randomly assigned to start as the agent. After reading the information sheet and listened to the instructions about the experiment, he said that he did not want to be the agent first but that the role of the victim was suitable for him. The other participant agreed to start as the agent. When arrived the time of role reversal, he confirmed that he did not want to be in the role of the agent. During the debriefing, he reported that even if the shocks were not very painful, he felt uncomfortable with the idea to deliver a shock to the co-participant without any other reasons than the monetary reward.<br><br>(3) The third participant started as agent. He started with the free-choice condition and did not administer shocks to the 'victim'. In the coercion condition, the first time that the experimenter told him to deliver a shock, he pressed the other key. The experimenter thus told him to follow the instruction that was given. The next time the experimenter told him to deliver a shock, he again pressed the other button. We (the experimenter and the main investigator who were both present in the room) asked him if he wanted to continue this experimental condition and he told us not because he did not want to deliver a shock. He added that he did not want to stop the entire

experiment and asked to continue as 'victim'. The other participant thus turned agent and followed instructions in the coercion condition. During the debriefing, he reported that he felt uncomfortable with the idea to deliver a shock to the co-participant in exchange for money.

Randomization	Participants were assigned non-randomly to the different groups of individuals tested, according to their military rank or civilian category (i.e. civilian students, junior cadets, senior cadets, and privates)

Reporting for specific materials, systems and methods

We require information from authors about some types of materials, experimental systems and methods used in many studies. Here, indicate whether each material, system or method listed is relevant to your study. If you are not sure if a list item applies to your research, read the appropriate section before selecting a response.

Materials & experimental systems

n/a	Involved in the study
☒	☐ Antibodies
☒	☐ Eukaryotic cell lines
☒	☐ Palaeontology
☒	☐ Animals and other organisms
☐	☒ Human research participants
☒	☐ Clinical data

Methods

n/a	Involved in the study
☒	☐ ChIP-seq
☒	☐ Flow cytometry
☒	☐ MRI-based neuroimaging

Human research participants

Policy information about studies involving human research participants

Population characteristics	See above
Recruitment	Participants were selected through advertisements based on voluntary participation within each group (civilians, junior cadets, senior cadets, privates). No selection biases were expected.
Ethics oversight	The study was approved by the local ethical committee of the Université libre de Bruxelles (permission 008/2016).

Note that full information on the approval of the study protocol must also be provided in the manuscript.
